# Effectiveness of a local recruitment registry in older adults in Southern California

**DOI:** 10.1186/s12874-025-02640-z

**Published:** 2025-07-31

**Authors:** Adam I. Birnbaum, Adrijana Gombosev, Zion T. Grant-Freeman, Dan Hoang, Daniel L. Gillen, Joshua D. Grill

**Affiliations:** 1 Department of Statistics, University of California, Irvine, Bren Hall 2019, Irvine, CA 92697-1250 USA; 2 Institute for Memory Impairments and Neurological Disorders, University of California, Irvine, Irvine, CA USA; 3 Department of Neurobiology and Behavior, University of California, Irvine, Irvine, CA USA; 4 Department of Psychiatry and Human Behavior, University of California, Irvine, Irvine, CA USA

**Keywords:** Registries, Recruitment, Research attitudes questionnaire, Clinical trials, Alzheimer’s disease

## Abstract

**Background:**

Despite their potential as tools for improving the efficiency of accrual in clinical research, few analyses have assessed the effectiveness of recruitment registries. We investigated referrals to clinical research studies from the University of California, Irvine Consent-to-Contact registry, a local online recruitment registry in Orange County, CA. Our analyses sought to quantify the total number of referrals and the proportion of referrals leading to subsequent enrollment in a clinical research study. Key secondary objectives were to investigate the relationship with referral outcomes for registrant research attitudes and the participant burden of the contacting study.

**Methods:**

We categorized referral outcomes as: (i) unable to reach, (ii) declined to participate, (iii) phone screened but ineligible, (iv) consented but ineligible, or (v) consented and enrolled. We assessed the overall effectiveness of the registry using the observed distribution of referral outcomes. We investigated the marginal relationship between referral outcome and the participant burden of the contacting study using distributions of referral outcomes conditional on participant burden. We used a binary logistic linear mixed effects model to investigate relationships between registrant characteristics and the odds of a referral leading to participant consent.

**Results:**

Overall, the registry made 6,314 resolved referrals of 3,843 unique registrants to 57 unique research studies. 1,642 (26%) of these referrals were successful, leading to enrollment. Among referrals to interview studies, registrant research attitudes as measured by the research attitudes questionnaire were not associated with the odds of successful first referral (odds ratio per 5 points: 1.00, 95% CI: (0.79, 1.28), *p* = 0.98). Registrant research attitudes were positively associated with the odds of a successful first referral in non-invasive longitudinal studies (odds ratio per 5 points on the research attitudes questionnaire: 1.17, 95% CI: (1.03–1.32), *p* = 0.02). The observed relationship was stronger, though not significant in invasive and interventional research (odds ratio: 1.22, 95% CI: (0.99–1.49), *p* = 0.06).

**Conclusions:**

Our results emphasize the potential effectiveness of recruitment registries and of the research attitudes questionnaire to predict referral outcomes to higher burden clinical research.

## Background

Clinical research faces myriad barriers to successful recruitment [1–5]. In Alzheimer’s disease and related dementias (ADRD) clinical research, for example, the need for dyadic enrollment and possibility of requiring invasive procedures such as PET imaging and lumbar puncture limit the pool of eligible and willing participants [1, 2, 6, 7]. In all disease domains, meeting enrollment goals efficiently is crucial for researchers to ensure that their studies are adequately powered and capable of answering scientific questions of interest.

Recruitment registries have been proposed as an avenue for improving the efficiency of accrual. The promise is that registries can alleviate some of the logistical burden of recruitment by giving study teams the ability to contact a large number of potential participants quickly. Additional demographic data collected by registries can be used by recruitment teams to prioritize outreach to registrants who are more likely to be eligible or willing to participate [8].

There has been a large investment in registries as tools for facilitating study accrual [9–14]. In fact, in ADRD, NIH-funded Alzheimer’s Disease Research Centers (ADRC) are strongly encouraged to maintain an accompanying registry in addition to their longitudinal clinical cohorts [15]. Despite this investment, limited data are available on whether and how registries achieve their ultimate goal. The University of California, Irvine (UCI) Consent-to-Contact (C2C) registry is an online local recruitment registry launched in 2016 [16]. We sought to investigate the effectiveness of the C2C as a recruitment tool. Operated by investigators from the UCI ADRC, the registry has emphasized older adults, but is open to all individuals age 18 or older and has been made available to investigators conducting clinical research in any area [8].

This study had three goals. We first aimed to quantify the total number of referrals made from the C2C registry and how often they led to enrollment in recruiting studies. Second, we assessed the relationship between the participant burden of the recruiting study and referral outcomes, hypothesizing that higher burden studies would have lower referral success probabilities. Third, we sought to investigate associations between registrant characteristics and the probability of a referral leading to enrollment. We hypothesized that general biomedical research attitudes as measured by the 7-item research attitudes questionnaire (RAQ) are positively associated with the odds of a successful referral and that this association may be modified by the participant burden of the recruiting study.

## Methods

### The Consent-to-Contact registry

The C2C is an online local recruitment registry primarily enrolling residents of Orange County, CA and referring them to studies at UCI. Multiple design features of the C2C are aimed at increasing the likelihood that referrals lead to subsequent enrollment. Upon joining the registry, C2C registrants are asked to fill out a survey to collect demographics, family disease history, medical history including prescription medications, and lifestyle information (e.g., diet and exercise habits using validated instruments) [16]. Although some features of the C2C were designed around recruiting to preclinical AD trials, the registry was designed to be used for studies in any research domain.

### Sample of C2C registrants

We used data on C2C referrals from the registry’s launch on August 16, 2016, through January 26, 2024. Some registrants were referred but subsequently withdrew consent to participate in the C2C. The outcomes of these referrals are included where possible but omitted from analyses that required individual-level data. To assess the overall effectiveness of the C2C, we used all referrals with an observed outcome (*N* = 3,843 unique registrants, *n* = 6,314 referrals). 250 referrals were shared with recruiting investigators but were not resolved as of January 26th, 2024. Fifty-two referrals were made, but recruitment closed before registrant responses were determined. To assess the association between study burden and enrollment outcome we used registrants’ first referral. Sixteen C2C registrants had ambiguity as to which among multiple study teams were the first to contact them. For these 16 registrants, we randomly sampled among the possible first referrals. Due to the low number of ambiguous first referrals relative to the overall sample size, sensitivity of the reported results to this imputation is trivial. To assess the association between the odds of a successful first referral (see below) and the RAQ, we performed a complete case analysis (*N* = 3,467 subjects).

### Defining referral outcomes, research willingness, and study burden

Referral outcomes were coded as: (i) unable to reach, (ii) declined to participate, (iii) phone screened but ineligible, (iv) consented but ineligible, or (v) consented and enrolled. We treated any referral leading to participant consent as successful. Registrants in the C2C are asked to report their willingness to be contacted about studies involving the following: (i) taking an approved medication, (ii) taking an investigational medication, (iii) altering diet or lifestyle, (iv) blood draws, (v) cognitive testing, (vi) MRI, (vii) PET scan, (viii) lumbar puncture, and (ix) autopsy. Registrants are never referred to studies involving procedures they have not indicated willingness to consider. We collapsed the willingness construct into a factor with three levels: 0–5 affirmative indicators were coded as low, 6–7 as medium, and 8–9 as high willingness. This was done a priori based on prior work investigating individuals’ willingness to undergo these procedures [6, 7, 16]. We coded studies receiving referrals according to their participant burden as: low (interview studies), moderate (non-invasive longitudinal studies), or high (invasive and interventional research). Two independent raters (ZTG and AG) assigned the burden level to all studies and a third rater (JDG) settled any discordances.

### The research attitudes questionnaire

The RAQ is a short 7-item survey that assesses general attitudes toward biomedical research and was designed in part to be predictive of willingness to participate [17]^,^ [18]. Each item is scored on a scale from 1 to 5 for a total score between 7 and 35. Higher scores indicate more positive attitudes toward research.

### Statistical analyses

To investigate the overall effectiveness of the C2C, we assessed the empirical distribution of referral outcomes. To investigate the marginal association between study burden and the outcome of first referrals, we used the distributions of outcomes conditional on burden level. We estimated the association between the RAQ and the probability of successful first referral via a logistic generalized linear mixed effects model (GLMM). We included study-specific random intercepts to account for heterogeneity in the baseline probability of acceptance across different studies and for potential correlation between referrals to the same study. Along with the RAQ, we controlled for the following potential confounding factors and precision variables: age, sex, race & ethnicity, years of education, research willingness, and study burden. We included an interaction effect between the RAQ and study burden. Model covariates and hypotheses were specified a priori. We performed an imputation-based sensitivity analysis to assess the impact of missing data on modeling results. As a secondary analysis we fit an analogous GLMM modeling RAQ as a binary construct of either high (≥28) or low (< 28), drawing on prior work suggesting 28 as a meaningful cutpoint for predicting research behaviors [19, 20].

Our primary inferential targets were the linear contrasts of model coefficients defining the relationship between the RAQ and odds of successful first referral within each study burden stratum. We conducted an omnibus test of the null hypothesis that all RAQ-related model coefficients are equal to zero; for this we used a likelihood ratio test. All other P-values were obtained via 2-sided Wald tests. We performed our analyses using R version 4.2.2 and used the ‘lme4’ package for fitting the GLMM.

## Results

**Fig. 1:**
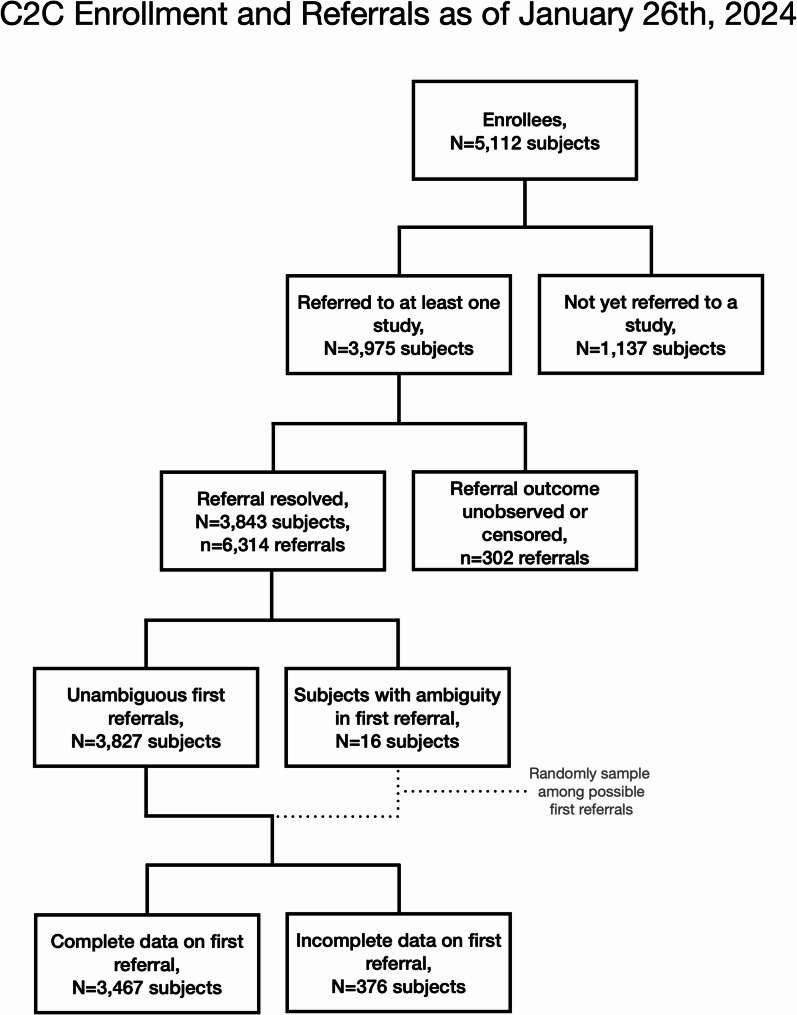
C2C Enrollment and Referrals Flowchart

The C2C made 6,314 resolved referrals of 3,843 unique registrants to 57 unique research studies during the study period (Fig. 1). Among these studies, 6 were low burden, 33 were moderate burden, and 18 were high burden. Table 1 provides the empirical distribution of the referral outcomes. 1,642 (26%) of the referrals led to consent. These successful referrals were distributed among 1,313 unique registrants; 1,024 of these registrants enrolled in a single study, 250 enrolled in two studies, 38 enrolled in three studies, and a single registrant enrolled in four studies. In 42.5% of referrals the recruiting study team was unable to reach the registrant and in 21.1% of referrals the registrant declined to participate. Only 1.6% of referrals led to a study obtaining consent from a registrant subsequently found to be ineligible.

**Table 1:** Empirical distribution of referral outcomes in the C2C

	Count (%)
Unable to reach	2,681 (42.5)
Declined to participate	1,334 (21.1)
Phone screened & ineligible	657 (10.4)
Consented & ineligible	102 (1.6)
Consented & enrolled	1,540 (24.4)
Total	6,314

Table 2 provides the demographics of the C2C registrants referred to studies, along with the number of referrals and referral success rate within applicable demographic strata. Nearly two-thirds of referred C2C registrants were women and just over three-quarters were Non-Hispanic White. Over 90% had more than 12 years of education. Nearly 60% of referred registrants indicated high levels of research willingness.

**Table 2:** Demographic information on C2C registrants who received a resolved referral; number of total referrals and referral success probability within each demographic stratum are given in in the two columns on the right

	Registrant characteristics, *N* (%) or mean (sd)	Total referrals, *N*	Success rate, %
Sex			
Male	1,344 (36.1)	2,293	23.4
Female	2,364 (63.5)	3,851	26.9
Other	13 (0.3)	13	15.4
Missing	122	157	42.0
Age at first referral	61.0 (15.6)	N/A	N/A
Missing	162		
Race and Ethnicity			
Non-Hispanic white	2,710 (75.2)	4,633	27.6
Hispanic (any race)	353 (9.8)	504	21.8
Non-Hispanic black	51 (1.4)	77	18.2
Non-Hispanic Asian	298 (8.3)	517	19.5
Other	190 (5.3)	266	16.2
Missing	241	317	29.7
RAQ	28.8 (4.4)	N/A	N/A
Missing	174		
Education			
12 or fewer years	291 (7.9)	447	17.9
12–16 years	1,937 (52.4)	3,123	25.8
More than 16 years	1,469 (39.7)	2,553	26.9
Missing	146	191	36.6
Willingness score			
Low willingness	495 (13.5)	709	24.0
Medium willingness	1,023 (27.8)	1,665	23.1
High willingness	2,161 (58.7)	3,729	27.3
Missing	164	211	33.2

The number of total referrals within each demographic substratum were roughly proportionate to the stratum size. In the observed sample, referrals of Non-Hispanic White registrants more often resulted in enrollment compared to referrals to registrants belonging to other racial and ethnic groups.

Table 3 provides the outcomes among first referrals stratified by study burden level. Low burden studies had the highest rates of enrollment at 45.8%. Moderate and high burden studies had meaningfully lower rates at 22% and 26.3%, respectively. Referrals to moderate burden studies also had a notably higher rate of being unable to reach the potential registrants than referrals to low or high burden studies (53.1% for moderate burden studies, 31.8% and 23.4% for low and high burden studies, respectively). Rates of registrants consenting but subsequently being found to be ineligible were similarly low across all study burden levels, although observed rates of being determined to be ineligible by phone were highest for high burden studies.

**Table 3:** Empirical distribution of first referral outcomes stratified by study burden

	Low burden	Moderate burden	High burden
Unable to reach	148 (31.8)	1320 (53.1)	209 (23.4)
Declined to participate	86 (18.5)	376 (15.1)	304 (34.0)
Phone screened & ineligible	18 (3.9)	241 (9.7)	145 (16.2)
Consented & ineligible	7 (1.5)	30 (1.2)	10 (1.1)
Consented & enrolled	206 (44.3)	518 (20.8)	225 (25.2)
Total	465	2485	893

**Fig. 2:**
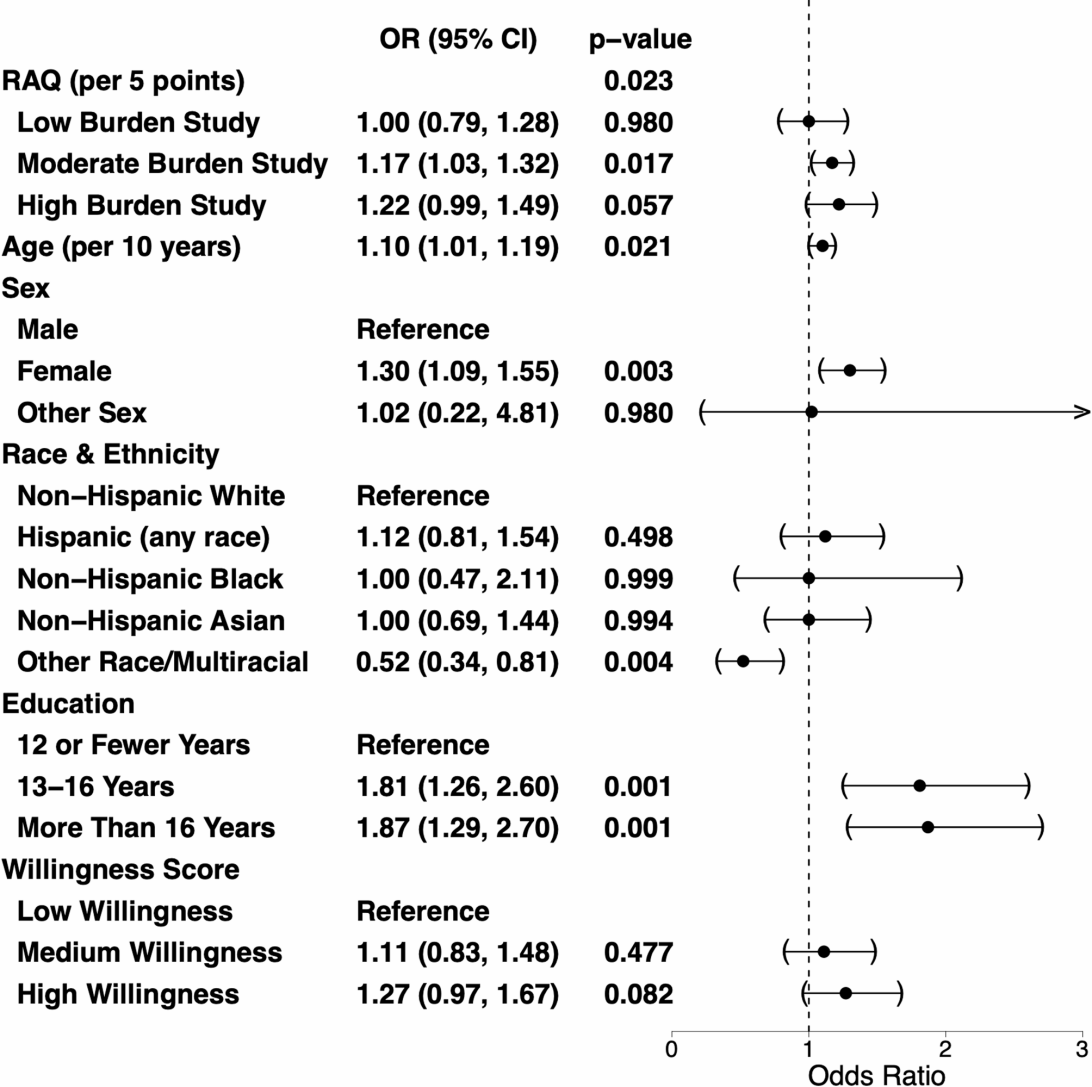
Left: Tabular summary of inference on fixed effects in binary logistic generalized linear mixed-effects model; Right: Forest plot of ORs and 95% CIs; RAQ: research attitudes questionnaire; OR: odds ratio; CI: confidence interval

Figure 2 contains a tabular summary and forest plot of estimated odds ratios (ORs) and 95% confidence intervals (CIs) from the binary logistic GLMM. The omnibus likelihood ratio test of all RAQ-related model coefficients indicated a significant association between the RAQ and the odds of a successful first referral overall (*p* = 0.02). In low burden studies there was no evidence of an association between the RAQ and the odds of a successful first referral (OR: 1.00, 95% CI: (0.79,1.29), *p* = 0.98). In moderate burden studies, higher scores on the RAQ were associated with higher odds of a successful first referral (OR: 1.17, 95% CI: (1.03, 1.32), *p* = 0.02). In high burden studies, we observed 22% increased odds of a successful first referral per 5 points on the RAQ, though this was not significant at the 0.05 level (OR: 1.22, 95% CI: (0.99, 1.49), *p* = 0.06).

When repeating the analysis using the binary construct of RAQ, we observed a similar trend in moderate burden studies although the CI crossed over one (high RAQ: OR: 1.20, 95% CI: (0.96, 1.50), *p* = 0.11). In high burden studies the association was attenuated (high RAQ: OR: 1.04, 95% CI: (0.72, 1.50), *p* = 0.82). In low burden studies, we estimated that referrals to registrants with RAQ scores 28 or higher had 12% higher odds of success compared to similar registrants with RAQ scores less than 28, but the result was not statistically significant (high RAQ, OR: 1.12, 95% CI: (0.72, 1.75), *p* = 0.61).

We also estimated that female registrants had 30% higher odds of a successful first referral, compared to similar male registrants (95% CI: (1.09, 1.55), *p* = 0.003). Education was also associated with odds of successful first referral, with registrants having 12–16 or more than 16 years of education having around 80% higher odds of a successful first referral compared to similar registrants who had fewer than 12 (13–16 years: OR: 1.81, 95% CI: (1.26, 2.60), *p* = 0.001; more than 16 years: OR: 1.87, 95% CI: (1.29, 2.70), *p* = 0.001). Overall, we saw little differences across racial and ethnic groups although those in the other race/multiracial group had significantly lower odds of successful first referral compared to similar Non-Hispanic White registrants (OR: 0.52, 95% CI: (0.34, 0.81), *p* = 0.004). The sensitivity analysis suggested missing data had little impact on qualitative conclusions.

## Discussion

Recruitment registries are an innovative tool to alleviate the challenges in study recruitment by assembling a pool of potentially willing and eligible study participants. We investigated the outcomes of study referrals in a local recruitment registry and found this tool to be effective in supporting accrual to a large number of studies of varying levels of participant burden.

Overall, the C2C registry made 6,314 referrals of 3,843 unique individuals to 57 studies during the reporting period. Over 1,600 of these were successful and nearly 300 of these referrals were to high burden research, which we expect to be more difficult to recruit to due to requiring potentially invasive procedures [6, 7]. Referrals leading to in-person screen failure were relatively few (~ 2%), providing evidence that the approach of referring registrants only to studies for which they are preliminarily eligible may maximize registry efficiency. Still, the C2C registrant data are limited and cannot account for all study inclusion criteria; this limitation may be reflected by the 10.4% phone screen fail rate, and that this rate was highest for high burden research (which likely has the most restrictive eligibility criteria). This finding may instruct the difficult balance that registry designers must strike between the length of time it takes to enroll in a registry and peoples’ willingness to enroll in it [21]. Designers would be prudent to focus on collecting features most relevant to common research eligibility criteria.

Marginally, first referrals to low burden studies had nearly twice the success rate of moderate or high burden studies. This matched our expectations since, even accounting for the fact that those referred to higher burden studies indicated a potential willingness to undergo the procedures they include, invasive and interventional research asks more of participants in terms of commitment and risk. We observed a substantially higher rate of being unable to reach potential participants in moderate burden studies compared to low and high. We assume this is mostly a random occurrence although registrants referred to higher burden studies, who would have indicated higher willingness to undergo invasive procedures, might be the most motivated and therefore most likely to actively decline invitations as opposed to not responding to a referral they do not plan to accept.

We found evidence of a positive association between RAQ score and the odds of a successful first referral overall and in moderate burden studies, where the odds of a successful first referral were 17% higher per 5 points on the RAQ. In high burden studies, we observed a 22% increase in the odds of a successful first referral per 5 points on the RAQ, though we note this observed effect failed to reach significance at the 0.05 level. Future work might further investigate the relationship between the RAQ and referral success in high burden research. No evidence of an association was observed for low burden studies. This may suggest that research attitudes are most relevant to decision-making in studies that carry meaningful logistical burden and/or perceived risk. To our knowledge, this study is the first to investigate whether the RAQ associates with registry referral outcomes. This is unique empirical data that fits with other studies of research behaviors [19]. Operationally, these findings support the RAQ as a useful low-cost tool for identifying potential registrants most likely to accept invitations to participate. Future research should investigate whether intervention can increase RAQ scores and whether this translates to increased willingness to participate.

Prior analyses of data from the C2C found that individuals from historically underrepresented racial and ethnic groups were less likely than their Non-Hispanic White counterparts, on average, to indicate willingness to be contacted about studies involving the 9 research procedures [6]. We saw no effect of race and ethnicity on the outcome of first referral. Although seemingly discordant with the prior findings, these findings should be interpreted while also considering that (a) C2C registrants are referred only to studies that match their willingness preferences and (b) we controlled for composite willingness score. Our results therefore suggest that conditional on similar overall willingness and research attitudes, registrants from underrepresented racial and ethnic groups have similar odds of enrolling in a research study as their Non-Hispanic White counterparts. Thus, a crucial goal for registries should be to increase diversity and overall size to permit prioritization of referring diverse registrants who are willing to enroll in specific studies. Beyond initial registry enrollment, strategies for retaining these registrants is also essential as certain subpopulations have been found to be less likely to be retained in online recruitment registries [22, 23].

Other factors predictive of first referral outcome were sex and years of education: female C2C registrants had 30% higher odds of a successful first referral compared to similar male registrants. Those with more than 12 years of education had over 80% higher odds of a successful first referral compared to similar registrants with 12 or fewer years. The observed effects were not simply due to more positive research attitudes or greater willingness to participate in research in more educated subpopulations since we controlled for these factors. Future work should attempt to better understand barriers to achieving a successful referral for men and those with 12 or fewer years of education not related to overall research attitudes.

Our study had limitations. The C2C is an online local registry primarily serving Orange County, CA. Findings related to demographics of registrants and other results may not generalize to other geographic regions or larger registries. We did not have the exact date studies contacted registrants and used the date that their contact information was shared with a study as a proxy. Although likely infrequent, we cannot rule out the possibility that some referrals were coded as a registrant’s first when a separate study who received their contact information later ended up contacting them earlier. For estimating the association between the RAQ and the odds of a successful first referral, we attempted to control for potential confounders but there remains a possibility that we were biased for target associations due to unmeasured confounding. We did not account for the methods investigative teams used to contact registrants (e.g., email vs. phone calls), though the operational requirements for the C2C include collecting preferred contact modes for registrants and mandating aligned approaches by recruiting study teams. Further, some of registrants had missing demographic data; these registrants were not included in the regression analysis. We might expect that registrants who make sure to complete data forms are the most motivated to participate in clinical research. We therefore cannot rule out the possibility that associations between registrant demographics and referral success probability may differ slightly in the complete data cohort relative to the overall population, though sensitivity analysis suggested our qualitative conclusions were not impacted by missing data. Individuals may provide an email address only without enrolling fully in the C2C registry. Such individuals (*n* = 3,119) can be made aware of studies but were not included in the current analyses, resulting in an underestimation of the total number of research participants achieved through the tool. Finally, this study did not inform on the relative impact of the registry on recruitment outcomes compared to more traditional recruitment approaches.

## Conclusions

Overall, our results emphasize the effectiveness of carefully designed local recruitment registries. Our findings support the potential use of registrant research attitudes as measured by the 7-item RAQ, a simple validated instrument, as a low-cost tool for identifying registrants most likely to accept invitations to participate, particularly in moderate and high burden research studies.

## Data Availability

The data used and/or analysed during the current study are available from the corresponding author on reasonable request.

## Abbreviations

AD: Alzheimer’s disease
ADRD: Alzheimer’s disease and related dementias
PET: Positron emission tomography
NIH: National Institutes of Health
UCI: University of California, Irvine
C2C: Consent–to–Contact registry
RAQ: Research attitudes questionnaire
GLMM: Generalized linear mixed–effects model
OR: Odds ratio
CI: Confidence interval
